# Ethylene represses jasmonate signaling to attenuate nicotine biosynthesis in *Nicotiana tabacum*

**DOI:** 10.3389/fpls.2026.1823409

**Published:** 2026-04-20

**Authors:** Yongqiang Ding, Yulong Gao, Lu Zhao, Wenjing Wang, Ahui Tong, Bingwu Wang

**Affiliations:** 1 Tobacco Breeding and Biotechnology Research Center, Yunnan Academy of Tobacco Agricultural Sciences, Kunming, China; 2 Yunnan Daguan Laboratory, Kunming, China; 3 Tobacco Research Institute, Chinese Academy of Agricultural Sciences, Qingdao, China; 4 Kunming Institute of Botany, Chinese Academy of Sciences, Kunming, China

**Keywords:** ethylene, nicotine biosynthesis, regulation, tobacco, transcriptome analysis

## Abstract

It has been known for many years that ethylene represses Jasmonic acid (JA)-induced nicotine biosynthesis. However, it is unclear whether ethylene (ET) alone can inhibit nicotine biosynthesis, and if so, how it achieves this. In this study, we found that ethylene alone can suppress nicotine accumulation and the expression of nicotine biosynthesis and transport genes in tobacco. We then performed transcriptome analysis to explore the underlying molecular mechanism. Results showed that over 6000 differentially expressed genes (DEGs) after 2, 4, 8 and 24 h treatment of ethephon, an ethylene-releasing compound, were identified. GO and KEGG enrichment analysis revealed that DEGs were enriched in pathways related to nicotine biosynthesis, including “pyridine nucleotide metabolic process” and “pyridine-containing compound metabolic process” 4 h after ethephon treatment. Further analysis revealed that expression of key regulators of JA-mediated nicotine biosynthesis was significantly altered by ethephon treatment. Specifically, CORONATINE-INSENSITIVE 1 (COI1) genes *COI1*/*COI1L* and ETHYLENE RESPONSE FACTOR (ERF) genes *ERF189*/*199*, which are two major positive regulators of nicotine biosynthesis, were downregulated, whereas four JASMONATE-ZIM-DOMAIN (JAZ) genes *JAZ1*/*2b*/*7a*/*10* were upregulated, indicating that ET suppresses nicotine biosynthesis, at least in part, by repressing the JA signaling pathway. In addition, we identified a set of ET-responsive candidate regulatory genes co-expressed with *ERF189*/*199*, providing a resource for future functional studies of nicotine biosynthesis regulation in tobacco roots.

## Introduction

Nicotine, the major pyridine alkaloid in *Nicotiana* species, can account for up to 3% of the dry weight in tobacco leaves and plays important roles in plant defense against insect herbivores. Interestingly, nicotine is not synthesized in tobacco leaves but exclusively in tobacco roots (Dawson, 1942). Once formed, it is transported via the vasculature to aerial tissues and sequestered in the vacuoles of leaf cells (Shoji and Hashimoto, 2011a).

Nicotine is composed of two heterocyclic rings, pyrrolidine and pyridine rings. The pyrrolidine ring is derived from ornithine or arginine through a four-step enzymatic pathway involving arginine decarboxylase (ADC), ornithine decarboxylase (ODC), putrescine *N*-methyltransferase (PMT), and *N*-methylputrescine oxidase (MPO) (Feth et al., 1986; Tiburcio and Galston, 1986; Hibi et al., 1994; Xu et al., 2004; Katoh et al., 2007). The pyridine ring originates from nicotinic acid, which is synthesized via the nicotinamide adenine dinucleotide (NAD) biosynthetic pathway catalyzed by aspartate oxidase (AO), quinolinic acid synthase (QS), and quinolinate phosphoribosyl transferase (QPT) (Wagner et al., 1986; Sinclair et al., 2000; Katoh et al., 2005). Finally, the pyrrolidine and pyridine rings are supposed to be condensed into nicotine under the action of two types of enzymes: orphan oxidoreductase A622 and berberine bridge enzyme-like (BBL) (Kajikawa and Hirai, 2009; Kajikawa et al., 2011).

The regulation of nicotine biosynthesis is influenced by development stages, environmental factors, and phytohormones (Baldwin, 1998; Shi et al., 2006; Li et al., 2007). Among the phytohormones affecting nicotine biosynthesis, jasmonic acid (JA) is the most extensively studied. It is well established that the active forms of JAs, such as MeJA, induce nicotine biosynthesis by binding to its receptor F-box protein CORONATINE-INSENSITIVE 1 (COI1), which is part of the E3 ubiquitin ligase complex SCF^COI1^, and then promote the degradation of JASMONATE ZIM-DOMAIN proteins (JAZs), which act as repressors of JA signaling pathway by binding and repressing the activity of MYC2, the master transcription factor involved in nicotine biosynthesis. Subsequently, liberated MYC2 stimulates nicotine biosynthesis by activating the expression of the nicotine biosynthesis genes, such as *PMT*, *QPT*, *A622*, and transport genes, such as multidrug and toxic compound extrusion (*MATE*) genes, and also the regulatory genes, such as ethylene responsive factor (ERF) gene *ERF199*, through binding to the G-box motif in the promoters of these genes (Shoji and Hashimoto, 2011b; Dewey and Xie, 2013; Sui et al., 2021).

Besides JA, it has been known that ethylene suppresses JA-induced nicotine biosynthesis. MeJA strongly induces *PMT* expression after 24 hours, but this induction is abolished when MeJA is co-treated with ethephon, a compound that releases ethylene *in vivo* (Shoji et al., 2000). The content of MeJA-induced nicotine in *Nicotiana attenuata* roots was strongly suppressed when ethephon was supplied (Kahl et al., 2000). These observations clearly indicate an antagonistic crosstalk between ET and JA in nicotine biosynthesis regulation. However, it is unclear whether ethylene alone can inhibit nicotine biosynthesis.

Activation of the ET signaling pathway begins with ET binding to its receptors, which leads to the inactivation of Constitutive Triple Response 1 (CTR1) and the subsequent dephosphorylation and cleavage of Ethylene Insensitive 2 (EIN2). The liberated EIN2 C-terminus (EIN2-CEND) indirectly enhances the activity of EIN3/Ethylene Insensitive-Like Protein1 (EIN3/EIL1), which are the key transcriptional regulators of ET response. Then, EIN3/EIL1 promote the transcription of numerous ethylene-responsive genes, including *ERF*s, such as *ERF1* (Solano et al., 1998; Nakano et al., 2006; Dubois et al., 2018). Several ERFs have been reported to be involved in nicotine production in tobacco. For example, ERF189 and its paralog ERF199 positively regulate nicotine biosynthesis by binding to GC-rich cis-elements in the promoters of downstream nicotine biosynthesis genes, such as *PMT* and *QPT* to upregulate their expression (Shoji et al., 2010; Qin et al., 2021). Some other ERFs, for instance, ERF91, ERF32 and ORC1 (also called ERF221), have also been shown to be positive regulators of nicotine biosynthesis (De Boer et al., 2011; Sears et al., 2014; Sui et al., 2019). Among these ERFs, ERF189 and ERF199 are the two major ERF regulators of nicotine biosynthesis, as simultaneous knockout of both genes results in ultra-low-nicotine tobacco (Shoji et al., 2020).

In this study, we tested whether ET alone is sufficient to reduce nicotine accumulation in tobacco and examined the associated transcriptional responses in roots, with particular attention to nicotine biosynthesis and JA signaling-related genes.

## Materials and methods

### Plant materials

Tobacco plants (*Nicotiana tabacum* L. cv. Y87) were cultivated under controlled conditions at 23 °C with a 14 h light/10 h dark photoperiod. Four-week-old tobacco plants grown in soil pots were treated with 50 μM ethephon, an ethylene-releasing compound to mimic exogenous ethylene treatment, by root irrigation twice per week. For transcript analysis, whole roots of tobacco plants were harvested at 0, 2, 4, 8, and 24 h after treatment, immediately frozen in liquid nitrogen, and stored until total RNA extraction. For nicotine measurement, leaf samples were collected 14 days after treatment. Three root or leaf samples were pooled to form one biological replicate, and three biological replicates were analyzed. A parallel set of plants treated with water was used as the control.

### Nicotine quantification

Leaf samples were dried at 105 °C for 20 min and then at 65 °C until completely dehydrated. The nicotine quantification was performed as described by Sui et al. (2019).

### Transcriptome sequencing and data analysis

RNA extraction, cDNA library construction, and sequencing were conducted by Beijing Novogene Co., Ltd. (Beijing, China) using the Illumina NovaSeq 6000 platform (Illumina, Inc., San Diego, CA, USA). Raw sequencing reads were processed with fastp (v0.23.1) to obtain high-quality clean reads. These reads were aligned to the *Nicotiana tabacum* L. reference genome using HISAT2 (v2.2.1). Only uniquely mapped reads were retained for subsequent gene expression quantification.

Read counts were generated using featureCounts in the Subread package (v1.5.0-p3). Differential gene expression analysis was performed with DESeq2, applying thresholds of false discovery rate (FDR) ≤ 0.05 and |log_2_(fold change)| ≥ 1 to identify differentially expressed genes (DEGs). Functional annotation and enrichment analyses of DEGs were carried out based on Gene Ontology (GO) and Kyoto Encyclopedia of Genes and Genomes (KEGG) databases. GO terms and KEGG pathways with corrected *P*-values ≤ 0.05 were considered significantly enriched.

### qRT-PCR assay

Total RNA was extracted with TRIzol^®^ reagent (Invitrogen, Carlsbad, USA) following the manufacturer’s protocol. First-strand cDNA was synthesized using a PrimeScript™ 1st Strand cDNA Synthesis Kit (Takara, Otsu, Japan) and employed as the template for quantitative real-time PCR (qRT-PCR). Amplification was carried out on a QuantStudio™ 5 Real-Time PCR System (Thermo Fisher Scientific, Waltham, USA) with GoTaq^®^ qPCR Master Mix (Promega, Madison, USA).

Gene-specific primers are listed in **Supplementary Table 1**. The *actin* gene was used as an internal control. Relative gene expression levels were calculated using the 2^–ΔΔCT^ method.

## Results

### Ethylene alone suppresses nicotine biosynthesis

**Figure 1:**
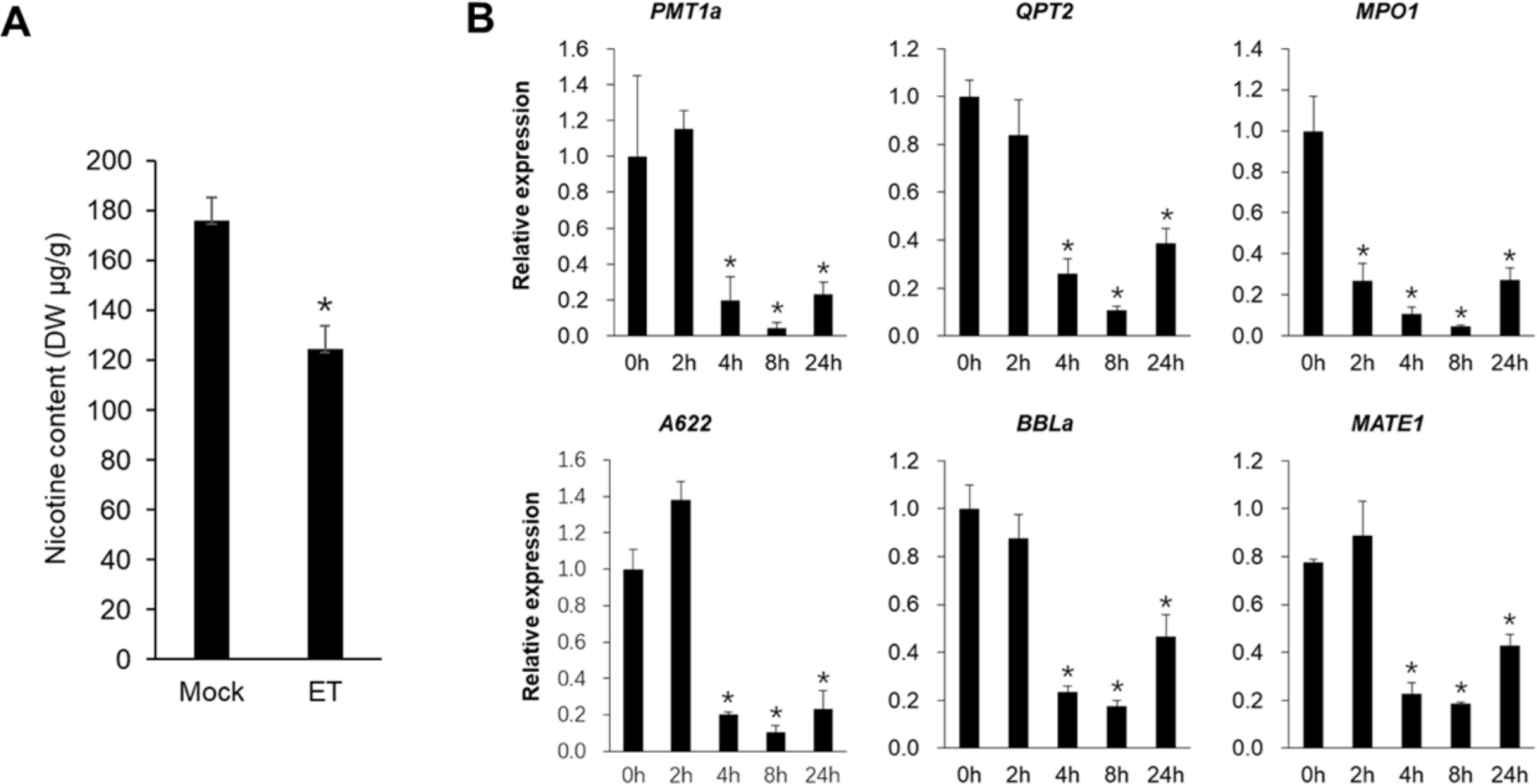
Ethylene suppresses nicotine biosynthesis in tobacco. **(A)** Nicotine content in tobacco seedlings after 14 days of ethephon treatment. **(B)** Relative expression of nicotine biosynthesis genes in roots of tobacco seedlings after indicated time points of ethephon treatment. Expression at 0 h was set to 1. Bars represent mean + SE of three biological replicates. Asterisk (*) indicates significant difference compared to 0 h (Student’s *t*-test, *P* < 0.05).

To investigate whether ethylene alone can suppress nicotine synthesis, tobacco seedlings were treated with 50 μM ethephon for 14 days. As shown in **Figure 1A**, the nicotine content of the leaves was significantly reduced by 30%, from 176 μg/g (dry weight, DW) in the control group to 124 μg/g DW in the ethephon-treated group.

We then analyzed the expression of key nicotine biosynthesis genes (*PMT1a*, *QPT2*, *MPO1*, *A622* and *BBLa*) and transport gene *MATE1* in the roots following ethephon treatment for 2, 4, 8, and 24 h. The transcript levels of these genes decreased significantly 4 hours after treatment (**Figure 1B**). These results demonstrate that ethephon treatment suppresses nicotine accumulation, accompanied by rapid downregulation of core biosynthesis and transport genes.

### Transcriptome analysis of ethephon-treated tobacco roots

**Figure 2:**
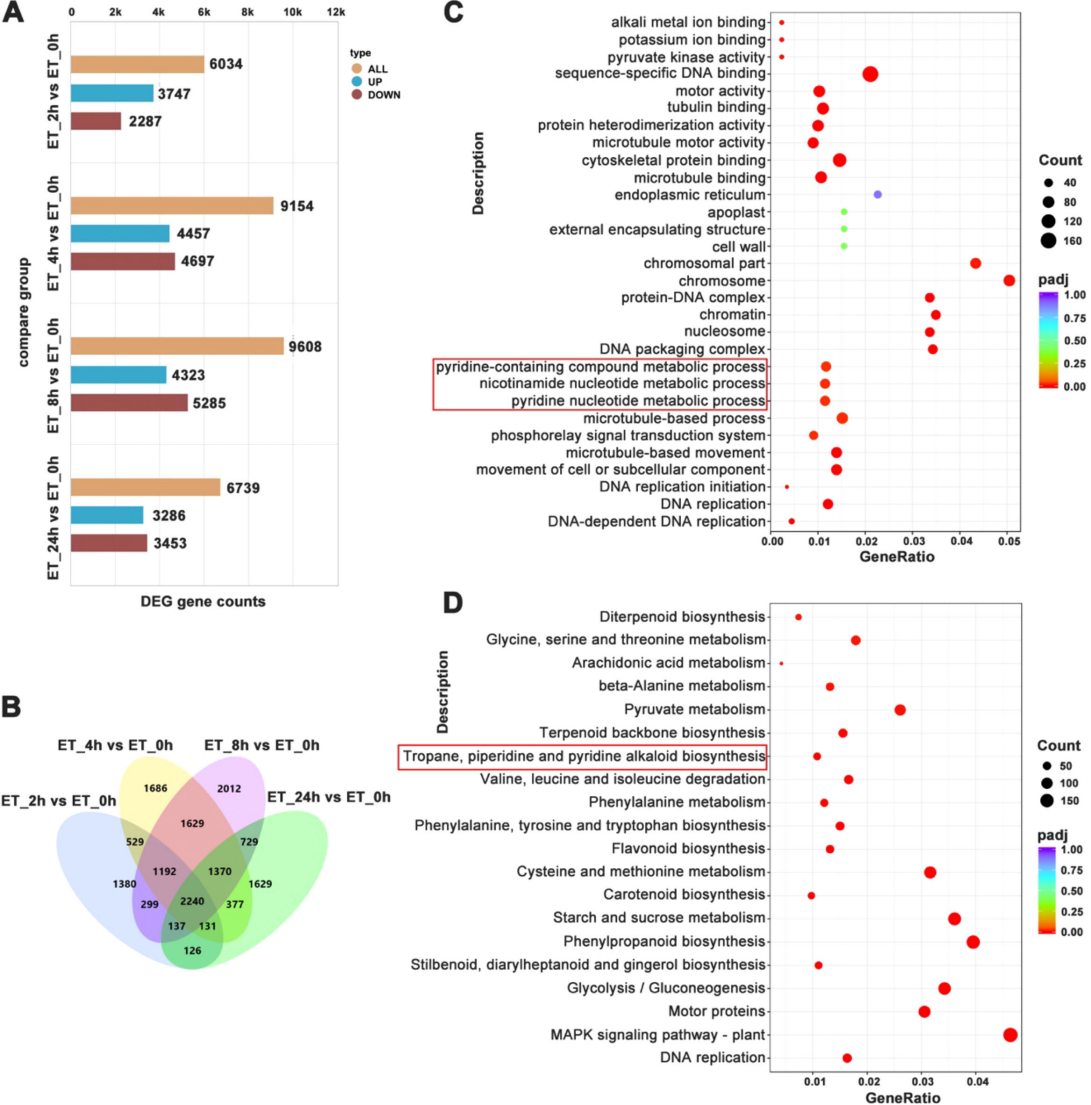
Analysis of differentially expressed genes (DEGs). **(A)** Graph of DEG numbers in indicated samples. **(B)** Venn plot of DEGs. **(C)** GO and **(D)** KEGG enrichment analysis of all differentially expressed genes following ethephon treatment for 4 h. Red boxes indicate GO terms or KEGG pathways related to nicotine biosynthesis.

Next, we performed transcriptome analysis on roots treated with ethephon for 2, 4, 8, and 24 h. Each sample yielded 52.61–76.90 million high-quality clean reads, with a mapping rate to the reference genome exceeding 90%. Pearson correlation analysis of biological replicates showed that the mean correlation coefficients within all treatment groups were above 0.9 (**Supplementary Figure 1**), indicating high reproducibility and reliability of the data. Using the 0 h sample as the control, we identified differentially expressed genes (DEGs) with the thresholds of adjusted *P*-value ≤ 0.05 and |log_2_ (fold change)| ≥ 1. The results revealed that upregulated DEGs were predominant at 2 h after treatment, whereas downregulated DEGs dominated at 4, 8, and 24 h. The number of DEGs increased over time, with 6,034, 9,154 and 9,608 genes identified at 2, 4 and 8 h respectively, and decreased to 6,739 at 24 h (**Figure 2A**), demonstrating that ethephon significantly reshaped the transcriptional profile in the roots. Venn analysis of the DEGs revealed 1380, 1686, 2012 and 1629 unique DEGs in roots at 2, 4, 8, 24 h after ethephon-treatment, respectively (**Figure 2B**).

GO and KEGG enrichment analyses were performed to elucidate the functional implications of transcriptional changes induced by ethephon. By 4 h, DEGs were significantly enriched in metabolic pathways involved in nicotine biosynthesis, including “pyridine nucleotide metabolic process” and “pyridine-containing compound metabolic process” (**Figures 2C, D**). Further KEGG analysis revealed that DEGs identified at 2, 8 and 24 h also showed consistent enrichment in the tropane, piperidine and pyridine alkaloid biosynthesis pathway, ranking among the top affected terms after treatment (**Supplementary Figure 2**). This persistent enrichment underscores the pronounced impact of ethylene on the transcriptional network governing alkaloid synthesis.

### Ethephon treatment altered the expression of genes related to nicotine biosynthesis

**Figure 3:**
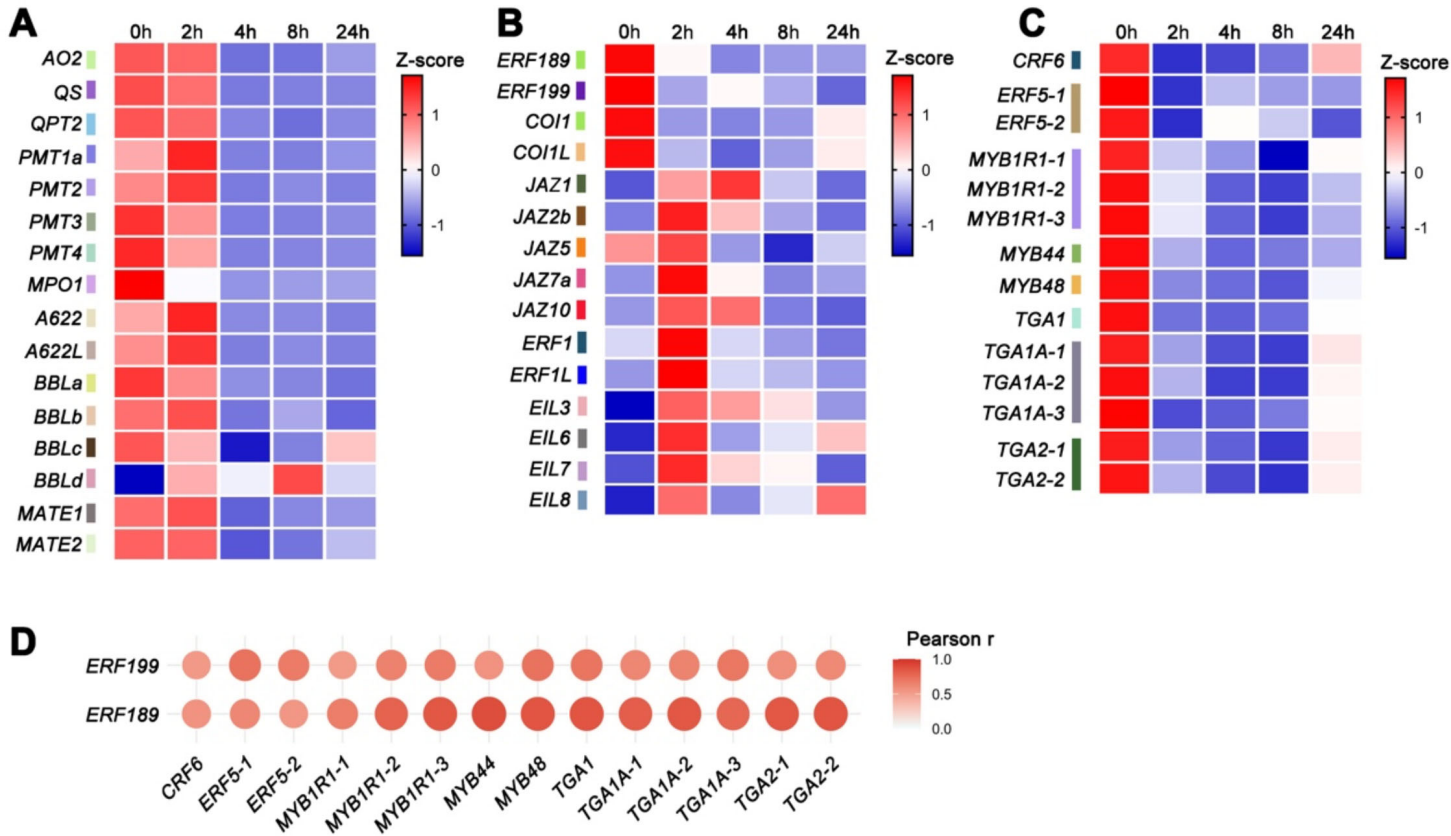
Expression profiles and correlation analysis of genes involved in nicotine biosynthesis, transport, and regulation in tobacco roots following ethephon treatment. **(A)** Heatmap showing the expression patterns of nicotine biosynthesis and transport genes, including *AO2*, *QS*, *QPT2*, *PMT1a*/*2*/*3*/*4*, *MPO1*, *A622*/*A622L*, *BBLa*/*b*/*c*/*d*, and *MATE1*/*2*, in tobacco roots at 0, 2, 4, 8, and 24 h after ethephon treatment. **(B)** Expression profiles of key genes in the JA and ethylene signaling pathways. **(C)** Expression patterns of candidate regulatory genes for nicotine biosynthesis. Specific gene identifiers are indicated for each row, consistent with the labels in C. Colors indicate relative expression levels shown as row-wise Z-scores. **(D)** Correlation analysis between the candidate regulatory genes and *ERF189*/*199*. Colors represent Pearson’s correlation coefficients, with red indicating positive correlation.

As shown in **Figure 3A**, nicotine biosynthesis and transport genes, including *AO2*, *QS*, *QPT2*, *PMT1a/2/3/4*, *MPO1*, *A622/A622L*, *BBLa/b/c* and *MATE1/2*, displayed a similar suppressed expression pattern, indicating coordinated suppression by ethephon. This finding is consistent with our qPCR data (**Figure 1B**).

Since the nicotine biosynthesis and transport genes are positively regulated by JA signaling pathway, we then analyzed the expression variation of the key components of JA pathway. For *COI1*, four paralogs were identified. Of the four *COI1* paralogs, two showed no significant change in expression, whereas the other two, *COI1*, a known positive regulator of nicotine biosynthesis (Shoji et al., 2008), and its paralog *COI1L*, were significantly downregulated as early as 2 h after ethephon treatment and remained repressed up to 24 h. Similarly, *ERF189* and *ERF199* were markedly suppressed by ethephon as well within the 2–24 h treatment. For *JAZ*s, a total of 25 transcripts were identified. Given the complexity of this gene family, we focused on the nine JAZs (JAZ1, JAZ2b, JAZ3, JAZ3b, JAZ5, JAZ7a, JAZ10, JAZ11b and JAZ12b) characterized to be able to bind to MYC2a and thus presumably involved in regulation of nicotine biosynthesis (Yang et al., 2014). Except for *JAZ3*, *JAZ3b*, *JAZ11b* and *JAZ12b*, which did not show significant change in expression, *JAZ1*, *JAZ2b*, *JAZ7*a and *JAZ10* were significantly upregulated as early as 2 h after ethephon treatment. Of them, *JAZ1* which was proved to be a negative regulator in nicotine biosynthesis (Shoji et al., 2008), showed about 7-fold and 10-fold increases in expression at 2 and 4 h after treatment, respectively. However, *JAZ5* showed a significant decrease of expression at 8 h after treatment. It seems that different JAZ family members may respond differentially to ethephon treatment. Meanwhile, we analyzed the effect of ethephon on the expression of *MYC2a*, which is known to be the master regulatory transcription factor in nicotine biosynthesis (Sui et al., 2021). The results showed that ethephon did not significantly affect *MYC2a* expression.

Since EIN3/EIL1, the essential components of ET signaling, were shown to interact with MYC2 in *Arabidopsis* (Song et al., 2014), we also examined the expression changes of the *EIN3/EIL1* orthologs in tobacco. Among the total 12 identified members of *EIN3*/*EIL1* family, except for eight of them with no significant alteration of expression observed, all the other four exhibited significant upregulation 2 h after ethephon treatment (**Figure 3B**). We further investigated the expression change of *ERF1* under ethephon treatment as well since it was reported that ERF1 inhibits JA signaling pathway (Chen et al., 2025). We found that the transcripts of *ERF1* and its paralog *ERF1L* were greatly induced 2 h after ethephon treatment, consistent with previous report that ET promotes the expression of *ERF1* (Solano et al., 1998) (**Figure 3B**).

### Fourteen candidate regulatory genes were identified

Through correlation analysis, we identified 14 transcription factor genes from eight different gene families (*ERF5*, *CRF6, MYB48*, *MYB44*, *MYB1R1*, *TGA1A*, *TGA2* and *TGA1).* These genes displayed similar expression patterns to *ERF189* and *ERF199* with Pearson’s correlation coefficients greater than 0.5 (*P*-value ≤ 0.05), suggesting that they may be involved in the ET-mediated suppression of nicotine biosynthesis (**Figures 3C, D**).

## Discussion

Nicotine, the predominant pyridine alkaloid in tobacco plants, plays an important defensive role against insect herbivores (Steppuhn et al., 2004; Dewey and Xie, 2013). Previous studies have demonstrated that several phytohormones, such as ET and JA, are involved in the regulation of nicotine accumulation (Shoji and Hashimoto, 2011a). Compared to JA, whose positive role in the regulation of nicotine biosynthesis has been well established (Dewey and Xie, 2013), the molecular basis of ET’s function in nicotine biosynthesis remains largely unknown, although a few studies reported that ET suppressed JA-induced expression of nicotine biosynthesis (Kahl et al., 2000; Shoji et al., 2000; Winz and Baldwin, 2001). To date, it remains unclear whether ethylene alone can inhibit nicotine biosynthesis.

In this study, we clearly showed that ET alone caused significant inhibition of the nicotine accumulation by suppression of nicotine biosynthesis and transport genes, including *PMT1a*, *QPT2*, *MPO1*, *A622*, *BBLa* and *MATE1* (**Figure 1B**). Then, we carried out transcriptome analysis of the root samples treated with ethephon to explore the underlying molecular mechanism of ET’s suppression role in nicotine biosynthesis. A total of 6034, 9154, 9608 and 6739 DEGs were identified at 2, 4, 8 and 24 h post-treatment, respectively. KEGG enrichment analysis revealed that DEGs were significantly associated with the nicotine formation related “tropane, piperidine, and pyridine alkaloid biosynthesis pathway” after ethephon exposure (**Figure 2**, **Supplementary Figure 2**). Except for the nicotine biosynthesis and transport genes, whose expression was significantly repressed by ethephon, we found that some key genes in JA-mediated nicotine biosynthesis pathway, including *COI1, COI1L* and *ERF189*/*199*, were significantly suppressed by ethephon as well. Conversely, among the nine JAZs that interact with MYC2a in tobacco (Yang et al., 2014), four of them, *JAZ1, JAZ2b*, *JAZ7a* and *JAZ10* were significantly upregulated, especially for *JAZ1* with an approximately 7-fold increase of expression 2 h after ethephon treatment. These results corroborate the previous findings that antagonistic cross-talk between ET and JA exists in the regulation of nicotine biosynthesis (Shoji et al., 2000; Winz and Baldwin, 2001) and indicate that ET suppressed nicotine biosynthesis, at least partially, by repressing the JA signaling pathway (**Figure 3B**). However, *MYC2a*, which is a master transcription factor in the JA-mediated nicotine biosynthesis (Sui et al., 2021), was not affected by ethephon treatment. Meanwhile, four *EILs*, which are orthologs of *EIN3*/*EIL1* in *Arabidopsis*, were also found to be significantly upregulated by ethephon treatment (**Figure 3B**). Previous studies showed that ET stabilizes EIN3/EIL1 protein, and EIN3/EIL1 interact with and repress MYC2’s transcriptional activation function, thereby attenuating JA-regulated defense response against herbivore attack in *Arabidopsis* (An et al., 2010; Song et al., 2014), we therefore speculate that in this study, upon ethephon treatment, the activity of MYC2a was inhibited by the upregulation of *EIL*s, resulting in reduced nicotine biosynthesis. In addition, *ERF1* and its paralog *ERF1L*, were shown to be significantly induced by ethephon (**Figure 3B**). Since ERF1 was reported to inhibit JA signaling pathway (Chen et al., 2025), it is reasonable to speculate that the upregulation of *ERF1*/*ERF1L* in this study would also contribute to the repression of JA signaling pathway.

On the other hand, we could not exclude the possibility that ET suppresses nicotine biosynthesis directly through EILs because one or two potential EIN3/EIL1 binding site(s) (EBS, ATGTAT) (Song et al., 2015) exist(s) in the promoters (about 2 kb upstream of the ATG start codon) of *ERF189*/*199*, *PMT1a*, *A622*, *JAZ1* and *COI1* (data not shown). To test this possibility, future work with knockout mutants of *EIL*s would provide in-depth information.

In addition, by correlation analysis, we identified 14 candidate genes (**Figures 3C, D**). These candidate genes represent excellent targets for future investigations into the regulation of nicotine biosynthesis.

## Data Availability

The datasets presented in this study can be found in online repositories. The names of the repository/repositories and accession number(s) can be found below: https://www.cncb.ac.cn, PRJCA047687.
