# Supplementary material for: Ethylene represses jasmonate signaling to attenuate nicotine biosynthesis in Nicotiana tabacum
Source: Front Plant Sci. 2026 Apr 20;17:1823409. doi: 10.3389/fpls.2026.1823409 (PMC13136284; doi:10.3389/fpls.2026.1823409)
Supplement: Supplementary file 1 [file DataSheet1.doc]

***Supplementary Materials:***


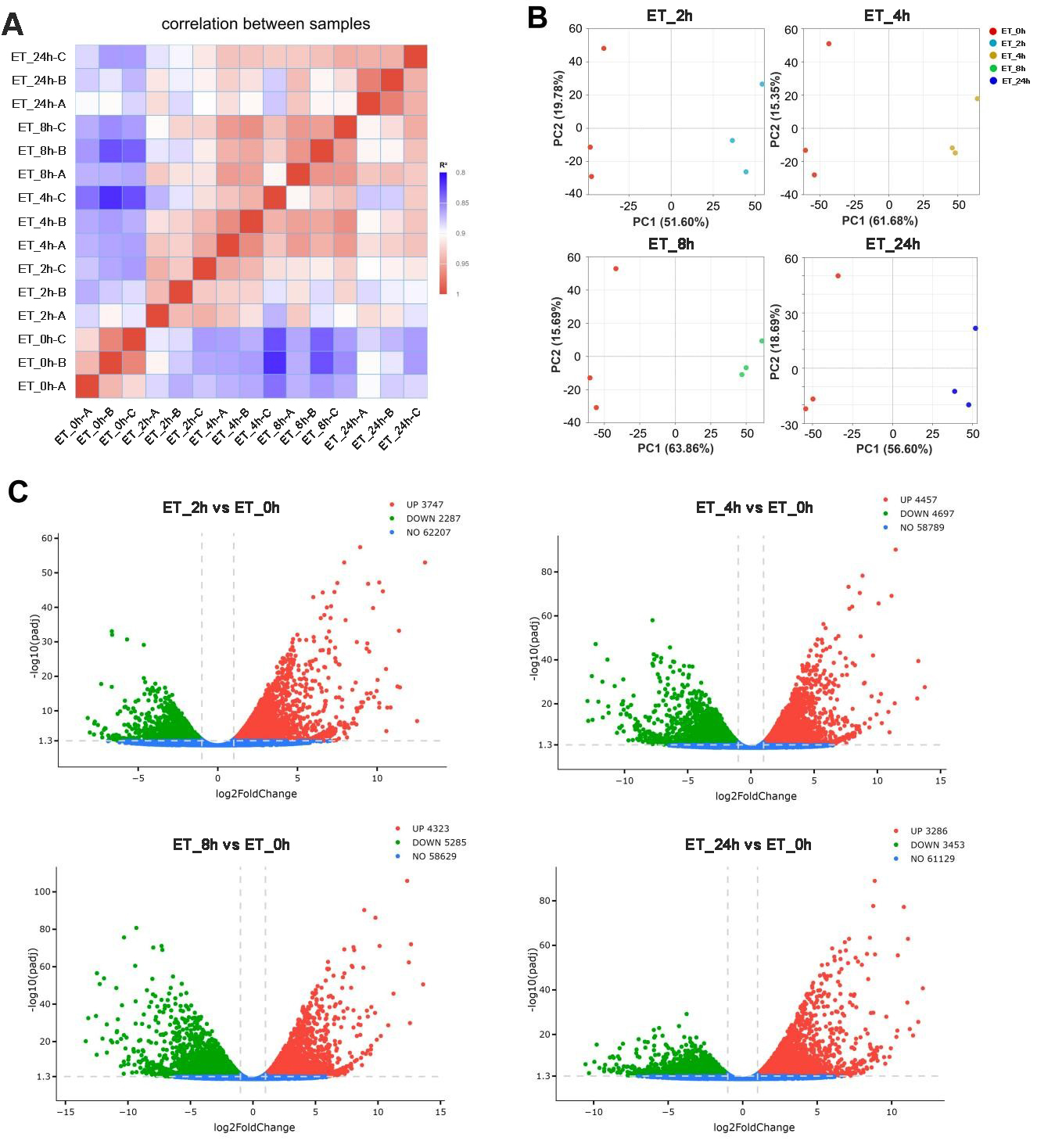


**Figure S1.** Transcriptomic analysis of genes differentially expressed in ethephon-treated tobacco roots. (**A**) Heatmap of correlation analysis between samples. The CK group comprised ET_0h_A, ET_0h_B, and ET_0h_C; the ET_2h group comprised ET_2h_A, ET_2h_B, and ET_2h_C; the ET_4h group comprised ET_4h_A, ET_4h_B, and ET_4h_C; the ET_8h group comprised ET_8h_A, ET_8h_B, and ET_8h_C; and the ET_24h group comprised ET_24h_A, ET_24h_B, and ET_24h_C. (**B**) PCA of transcriptomics data from tobacco roots treated with ET for 0 h, 2 h, 4 h, 8 h, and 24 h, respectively. (**C**) Volcano plots of DEGs.

**
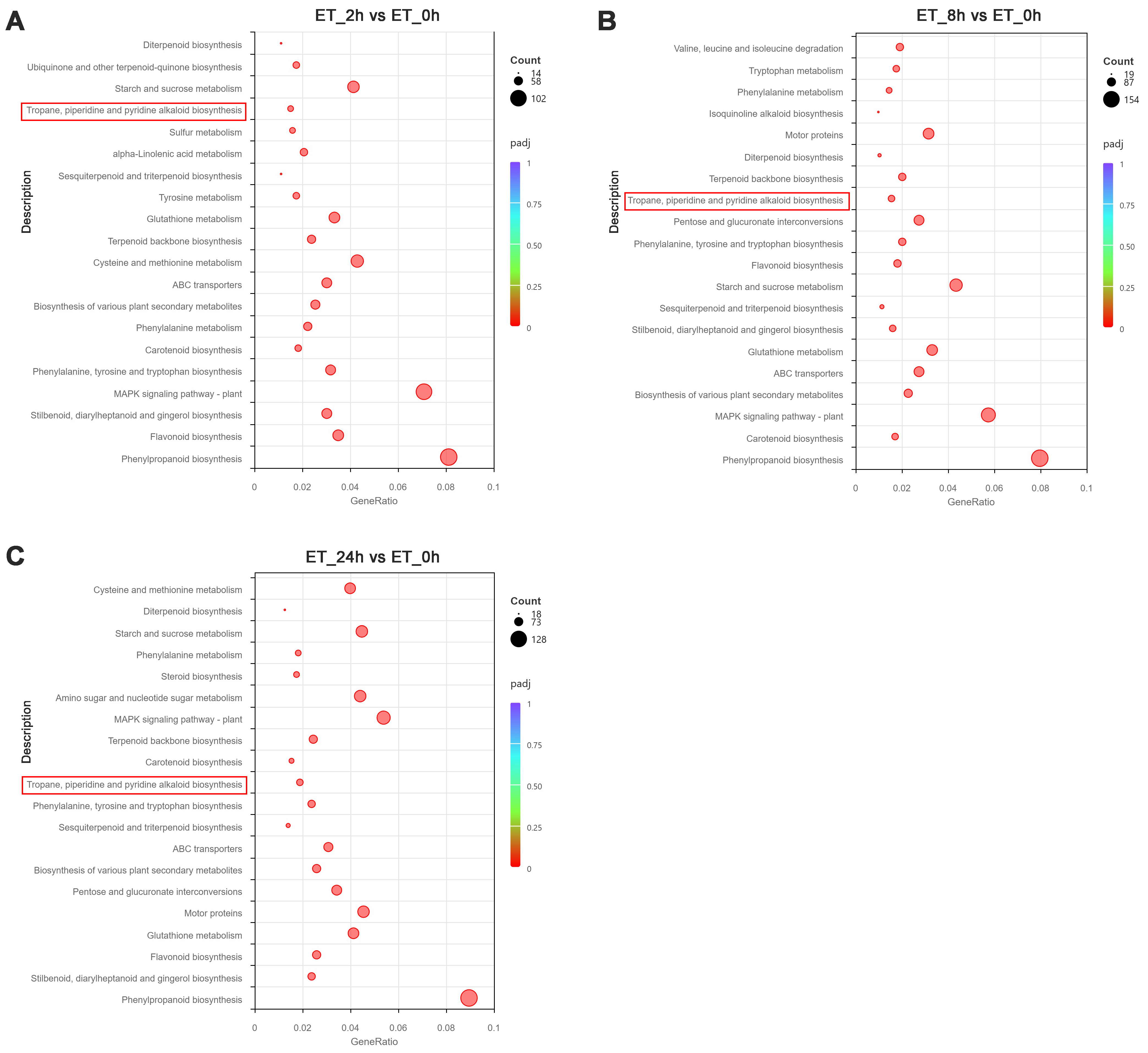
**

**Figure S2.** KEGG enrichment of DEGs following ethylene treatment for (**A**) 2 h, (**B**) 8 h, and (**C**) 24 h . Red boxes indicate KEGG pathways related to nicotine biosynthesis.

**Table S1** List of primers used for qRT-PCR analysis.

| qRT-PCR | *Actin*-F | CTGAGGTCCTTTTCCAACCA |
| --- | --- | --- |
|  | *Actin*-R | TACCCGGGAACATGGTAGAG |
|  | *PMT1a*-F | AAATGGCACTTCTGAACACCTC |
|  | *PMT1a*-R | CCCATTCTGGTGGCCGTTCC |
|  | *QPT2*-F | TACAAGAGTGGAGTCATTAGAG |
|  | *QPT2*-R | GCAAGTGCAATTCCTGCTATG |
|  | *MPO1*-F | CGATTTATTGAGGTGGTTCTGG |
|  | *MPO1*-R | GAAGCTTAGTAGGAATCTGAGAT |
|  | *A622*-F | GGATGATAGAGGCAGAAGGA |
|  | *A622*-R | TGACAACTTTGTCTCTAGGAG |
|  | *BBLa*-F | GATTTTACTCTAGGAGTACTGC |
|  | *BBLa*-R | TGTCTCATTCGATATGGAAAGA |
|  | *MATE1*-F | TGGCCAAAGAAGTATCTGAG |
|  | *MATE1*-R | TGTAACCCAAAGCAGAATAACA |
